# Another "Janus paradox" of p53: induction of cell senescence versus quiescence

**DOI:** 10.18632/aging.100165

**Published:** 2010-06-27

**Authors:** Zbigniew Darzynkiewicz

**Affiliations:** New York Medical College, Valhalla, NY 10595, USA

**Keywords:** 06/23/10; accepted: 06/25/10; published on line: 06/27/10

No other protein shows such multiplicity and diversity of functions as the tumor suppressor p53 [1, 2]. Initially, the role of p53 as "the guardian of the cellular genome" was considered to be providing protection from progression to malignancy. This was mediated by its function as a transcription factor of the genes controlling cell cycle and apoptosis [3]. Subsequent studies have identified a large variety of diverse genes regulated by p53. Among them are the genes modulating cellular senescence, DNA repair, oxidative stress, longevity, angiogenesis, differentiation, glycolysis, tumor motility and invasion, and even bone remodeling [1,2]. Independently of its transcription-regulatory mechanism p53 can also directly interact with proteins of Bcl2 family controlling the execution of apoptotic response [4].

It was recently reported that induction of cell senescence by ectopic expression of p21 and doxorubicin when combined with upregulation of p53 by inhibition of Mdm2, mediated by nutlin-3a, led to cell quiescence. The quiescence was reversible: upon removal of nutlin-3a the cells reentered the cell cycle [5]. This observation prompted the authors to postulate the use of Mdm2 antagonists in conjunction with chemotherapy to reversibly arrest normal cells, thereby protecting them from the drugs targeting cell cycle progression (cyclotherapy) [5]. Consistent with this observation were findings that p53 plays an important role in regulating stem cell quiescence, self-renewal and aging [6].

What is the mechanism by which p53 converts the cell response to the ectopic expression of p21 (cell cycle arrest) from senescence to quiescence? In recent studies Demidenko et al., addressed this question and in elegant experiments the authors demonstrated the "paradoxical" capabilities of p53, one to suppress cell senescence by inducing quiescence and another, already known, to induce senescence [3]. Suppression of senescence paralleled by induction of quiescence by p53 required its transactivation function, and in analogy to rapamycin, was mediated, at least in part, by inhibition of mTOR pathway [8]. Further evidence on the involvement of mTOR pathway in the direction the cell undertakes to become either senescent or quiescent is provided in the article in the current issue of Aging [9] consistent with their prior findings, the authors in this article report that induction of cell cycle arrest in the WI-38-tert or HT-1080-p21 cells, in which nutlin-3a inhibited mTOR, led to quiescence rather than senescence. In contrast, augmentation of mTOR pathway led to induction of senescence [9]. The data collectively suggest that in the process of induction of cells senescence or quiescence the primary role of p53 is in arresting cells in the cell cycle. However, the ongoing cell growth (rRNA synthesis) in the arrested cells mediated by mTOR pathway is the deciding factor as to whether they undergo senescence (mTOR activation) or quiescence (mTOR inhibition). The factor responsible for the apparent "paradoxical" properties of p53 was the dual and separate function of this protein, one arresting cells in cell cycle and another, inhibiting mTOR [7].

Senescent cells are characterized by large cell/nuclear size and "flattened" morphology, a characteristic feature of growth imbalance. It was shown before that cellular content of RNA (of which 95% is rRNA) in cycling cells is > 10-fold higher than in quiescent cells [10]. In contrast, the induction cell cycle arrest associated with the senescent phenotype is paralleled by several-fold rise in rRNA abundance [11]. It is also known that mTOR pathway regulates the synthesis of ribosomal components including the transcription and processing of pre-rRNA, expression of ribosomal proteins and the synthesis of 5SRNA [12]. The critical role of mTOR is thus in adjusting the ribosome biogenesis and overall protein biosynthetic capacity (cell growth) to the signaling through the growth factors pathway and coordinating it with the rate of cell cycle progression. Within this context cell senescence can be characterized as the uncoupling of the rate of cell cycle progression and cell growth mediated by mTOR. Of interest is the observation that mTOR activity is accelerated in hematopoietic stem cells from old mice compared to young mice prompting the authors to suggest that mTOR inhibitors can be used to rejuvenate aging hematopoietic cells [13].

Not disregarded should be a possibility of regulation of cell senescence by p53 via induction of autophagy. Here again the diverse "paradoxical" properties of p53 have been observed, namely the induction of autophagy upon activation and expression of this protein above the basal level and inhibition of autophagy after its induction to the basal level [14]. This "paradoxical" effect of p53, which on the surface appears to be contradictory, was metaphorically compared with the two-faced Roman mythology God and named The "Janus of Autophagy" [15]. Considering how extensively intertwined are the pathways of autophagy, senescence, apoptosis and aging the elucidation of the mechanisms involved in the induction of senescence versus quiescence by p53 is additionally complicated. Inhibition of mTOR while it enhances autophagy and thus is expected to delay senescence may also be lethal to cancer cells [16].  Further studies are needed to resolve how the "Janus of Autophagy relates to the "Janus of Cell Senescence" or to the "Janus of Cell Quiescence".
